# An efficient human stem cells derived cardiotoxicity testing platform for testing oncotherapeutic analogues of quercetin and cinnamic acid

**DOI:** 10.1038/s41598-022-21721-3

**Published:** 2022-12-09

**Authors:** Saurabh Mandal, Naisarg Gamit, Subhankar Biswas, C. Mallikarjun Rao, Gautam Sethi, Sudha Warrier

**Affiliations:** 1 Division of Cancer Stem Cells and Cardiovascular Regeneration, Manipal Institute of Regenerative Medicine, Manipal Academy of Higher Education (MAHE), Bangalore, 560 065 India; 2 Department of Pharmacology, Manipal College of Pharmaceutical Sciences, Manipal Academy of Higher Education (MAHE), Manipal, 576 104 India; 3 Department of Pharmacology, Yong Loo Lin School of Medicine, National University of Singapore, Singapore, 117 600 Singapore; 4 Cuor Stem Cellutions Pvt Ltd, Manipal Institute of Regenerative Medicine, Manipal Academy of Higher Education (MAHE), Bangalore, 560 065 India

**Keywords:** Cardiovascular diseases, Cell culture, Reverse transcription polymerase chain reaction, Fluorescence imaging, Cancer, Mesenchymal stem cells, Stem-cell biotechnology, Drug screening, Toxicology

## Abstract

Oncotherapeutics research is progressing at a rapid pace, however, not many drugs complete the successful clinical trial because of severe off-target toxicity to cardiomyocytes which ultimately leads to cardiac dysfunction. It is thus important to emphasize the need for early testing for possible cardiotoxicity of emerging oncotherapeutics. In this study, we assessed a novel stem cell-derived cardiac model for testing for cardiotoxicity of novel oncotherapeutics. We evaluated the cardiotoxic effect of synthesized derivatives of oncotherapeutics, quercetin (QMJ-2, -5, and -6) and cinnamic acid (NMJ-1, -2, and -3) using human Wharton's jelly mesenchymal stem cells-derived cardiomyocytes (WJCM) against known cardiotoxic oncologic drugs, doxorubicin, 5-fluorouracil, cisplatin. QMJ-6, NMJ-2, and NMJ-3 were not cardiotoxic and had minimum cardiac side effects. They did not show any effect on cardiomyocyte viability, caused low LDH release, and intracellular ROS production kept the calcium flux minimal and protected the active mitochondrial status in cardiomyocytes. They persevered cardiac-specific gene expression as well. However, compounds QMJ-2, QMJ-5, and NMJ-1 were cardiotoxic and the concentration needs to be reduced to prevent toxic effects on cardiomyocytes. Significantly, we were able to demonstrate that WJCM is an efficient cardiac testing model to analyze the cardiotoxicity of drugs in a human context.

## Introduction

Cancer is a multifactorial disease^1^ and due to tremendous advancement in treatment modalities such as surgery, chemotherapy, and radiation therapy, mortality has declined followed by improvement in survivorship^2^. However, cardiovascular diseases (CVDs) have emerged as major side effects among cancer survivors that may lead to long-term morbidity and premature deaths^3^. This is related to the outcome of cardiotoxicity resulting directly from the cancer treatment and that could have compromised the cardiac function and structure^4^.

Over time, a large number of the anti-cancer drug has been withdrawn from the market and the reason was an unintended effect on cardiac health^3^. The anthracycline class of chemotherapeutic drugs which consists of doxorubicin, 5-fluorouracil, paclitaxel, cisplatin, idarubicin, epirubicin, and mitoxantrone are known for exerting an adverse cardiotoxic effect on cardiomyocytes^5^. In 2016, the European Society of Cardiology (ESC) suggested a prior check to identify the risk of developing cardiovascular toxicity by any cancer treatments and therapy. Treatment modifications can thus be practiced that may allow decreasing the risk for further cardiotoxicity before administration to the patients^6^.

In search of a new therapeutic agent, chemically synthesized compounds whose structures mostly are inspired by natural compounds have been explored enormously against various disease models^7^. Bringing any newly synthesized anti-cancer molecule even after extensive preclinical testing, the entity faces several challenges to get translated for clinical utility because of their potential to cause cardiotoxicity^8^.

The heart comprises cardiac fibroblast cells, cardiomyocytes, endothelial cells, and vascular smooth muscle cells, and among them, cardiomyocytes are the main cells that carry out the vital function of pumping^9^. If an agent can induce cardiotoxicity, then it acts directly on cardiomyocytes, eventually leading to cardiomyocytes' death. There are potential moieties that include disruption of mitochondrial potential, reactive oxygen species (ROS) production, reduction in intracellular calcium level, and effect on signaling molecule, Nitric oxide (NO) level resulting in an adverse effect on cardiomyocytes. Simultaneously, cardiotoxicity reduces the metabolic activities and cardiac markers expression in cardiomyocytes^10,11^. Meanwhile, reliable cardiotoxicity testing models in vitro in the human context are lacking.

Traditionally, animal models have been used to evaluate the drug cardiotoxicity, which tends to be costlier and due to interspecies differences, the outcome remains uncertain^12^. With time, ethical concerns associated with the use of animals is getting bigger and the discussion to replace animal testing, reduce the number of animal use, and improve animal use practices has reached its peak^13,14^. Recognizing that the use of adult human cardiomyocytes isolated from biopsy or explanted heart tissues is not very feasible to maintain in culture^15^, the cell-based model has gained attention for the evaluation of drug cardiotoxicity at the preclinical stage.

There have been various in vitro screening approaches to assess the cardiotoxic effect of any drug molecule using cardiac cell lines and stem cell-derived cardiomyocytes^16^. Among stem cells, the ethical concern associated with the isolation of embryonic stem cells (ESCs) and teratoma formation ability by both the ESCs and induced pluripotent stem cells (iPSCs) still causes hindrance in their utility^17,18^. Apart from self-renewal property and multilineage differentiation ability, mesenchymal stem cells (MSCs) from prenatal tissues have additional features such as the presence of anti-aging regulatory factors and immunomodulatory properties^19,20^ and these traits of MSCs make it a potential alternative to ESCs and iPSCs. MSCs have been isolated from birth-associated tissues including the placenta, umbilical cord, Wharton’s jelly, cord blood, amniotic membrane, and amniotic fluid^21^. The placenta is mainly regarded as medical waste but it is a rich source of MSCs. One of the significant benefits of birth-associated tissues is their ready availability, non-invasive procedures of isolation, and they have no significant ethical and teratoma formation concerns^22^. Many research groups including ours previously demonstrated the cardiomyogenic differentiation potential of human Wharton’s jelly mesenchymal stem cells (WJMSCs). The prominent cardiac markers such as actin alpha cardiac muscle 1 (ACTC1), troponin T, Nkx2.5, and beta-myosin heavy chain (β-MHC) expression were found in differentiated cardiomyocytes^23–27^. Mechanistically, an epigenetic modification by the inducers directs the MSCs to differentiate into cardiomyocytes^28,29^.

Another alternative for animal use is H9C2 cells, an immortalized cell line derived from rat ventricular tissue. H9C2 cells are easily cultured, homogeneous cardiac cells population with rapid expansion property^30^. Several studies have considered H9C2 cells as a model for a wide range of toxicological studies due to their ability to respond to cardiac pathological stimulus, energy-related characteristics, glycolytic and energy metabolism, calcium handling, and electrophysiological properties^30–32^. However, considering the few obvious limitations^9,33^, we have included H9C2 cells to understand the preliminary differences in drug response between immortalized rat cardiomyocytes and primary human cardiomyocytes.

Besides primary and immortalized cardiomyocytes, human iPSCs-derived cardiomyocytes have gained attention over the last few years^34–37^. The hiPSC-derived cardiomyocytes exhibited major cardiac properties and have been able to recapitulate to different cardiac disease phenotypes^34,38–41^. Despite many advantages, hiPSCs are still far from perfect. The main challenges are the immature nature of cardiac cells which majorly emerges due to differences in induction cocktails, batch-wise variation in differentiation, and the reprogramming process itself may face setbacks due to undesired mutation or genomic instability^17,42^.

In this study, we constructed a cardiotoxicity testing model using cardiomyocytes derived from human Wharton’s jelly mesenchymal stem cells (WJMSCs) obtained from the umbilical cord to test the cardiotoxicity effect of two sets of novel oncotherapeutics which were derived from quercetin and cinnamic acid by Rao et al.^43–45^ which showed potent inhibition to colorectal cancer. We could observe cardiotoxicity and in some cases, lack of it using stem cell-derived human cardiomyocyte and rat H9C2 cardiomyoblast testing platforms.

## Material and methods

### Chemicals

Quercetin analogues (QMJ-2, QMJ-5, QMJ-6) and cinnamic acid analogues (NMJ-1, NMJ-2, NMJ-3) were synthesized in MCOPS, Manipal Academy of Higher Education, Manipal, India and previously reported as potent anti-cancer drugs against colorectal cancer^43,44^. Doxorubicin, 5-fluorouracil, and cisplatin were obtained from Sigma Aldrich, MO, USA.

### Cell culture

Mesenchymal stem cells from Wharton’s jelly (WJMSCs) were isolated from the Wharton’s jelly of the human umbilical cord via enzymatic digestion as described previously with slight modifications^23^. Experiments were performed after obtaining clearance from Institutional Ethical Committee (IEC), Manipal Hospital, Bangalore, India. Informed consent was obtained from the donor and all the experiments were performed in accordance with relevant guidelines and regulations. Rat cardiomyoblast cell line, H9C2 was obtained from National Centre for Cell Science (NCCS), Pune, India. WJMSCs were maintained in Knockout™ Dulbecco's Modified Eagle Medium/Nutrient Mixture F-12 (KO-DMEM/F-12; Gibco, MA, USA) with 10% Fetal bovine serum (FBS, Gibco, MA, USA), 1X GlutaMAX (Gibco, MA, USA), and 1X Antibiotic Antimycotic Solution (Gibco, MA, USA). H9C2 cells were cultured in Dulbecco’s Modified Eagle Medium–High Glucose (DMEM-HG; Gibco, MA, USA) supplemented with 10% FBS and 1X Antibiotic–Antimycotic solution at 37 °C in 5% CO_2_ incubator.

### Cardiomyogenic differentiation

Differentiation of WJMSCs was done using a proprietary combination of DNMT and HDAC inhibitors in KO-DMEM/F-12 containing 10% FBS for 48 h. Induction media was replaced with culture media i.e. KO-DMEM/F-12 with 1X GlutaMAX and, 10% FBS after 48 h for 7 days^23^.

### Treatments

WJMSC-derived cardiomyocytes (WJCM) and H9C2 were treated with NMJ and QMJ analogues for 24 h. The concentration of these compounds was based on their previously reported IC_50_ values which were obtained using Vero, a non-cancerous cell line i.e., QMJ-2 (140 µM), QMJ-5 (55.6 µM), QMJ-6 (153 µM), NMJ-1 (9.7 µM), NMJ-2 (8.2 µM), NMJ-3 (15.1 µM)^43,44^. To illustrate the toxicity specific to cardiac cells and not a general cytotoxicity, WJMSCs were treated with positive cardiotoxic drugs. The positive cardiotoxic drugs used were doxorubicin (Dox)^46^, 5-fluorouracil (5FU)^47^, and cisplatin (Cis)^48^.

### Cell viability assays

WJMSCs were treated with Dox (1 µM), 5FU (1 µM), and Cis (5 µM) for 24 h and then MTT and CCK-8 assays were performed. Similarly, the toxicity of these compounds in terms of the viability of cells was estimated by using the MTT assay and cell counting kit-8 (CCK-8) assay. For MTT assay, after 24 h of treatments, MTT (5 mg/ml of PBS; HiMedia, India) was added, and cells were incubated for 2 h at 37 °C. The formazan crystals that were formed after the incubation were dissolved using dimethyl sulfoxide (DMSO). For the CCK-8 assay, 10 μL of CCK-8 solution (Boster Biological Technology, Pleasanton, CA, USA) was added to each well and incubated for 2 h at 37 °C. The absorbance for MTT and CCK-8 assay was measured at 570 nm and 450 nm respectively using the EnSight™ Multimode plate reader (Perkin Elmer, MA, USA)^49,50^.

### Lactate dehydrogenase (LDH) assay

LDH activity was observed in WJMSCs treated with positive cardiotoxic drugs as well as in the H9C2 cells and WJCM after they were exposed to QMJ and NMJ analogues using Pierce LDH cytotoxicity assay kit (Thermo Scientific, MA, USA). The experiment was performed as per the manufacturer’s protocol. Absorbance was taken at 490 nm and 680 nm (background signal) using multimode plate reader. LDH activity was calculated by subtracting absorbance taken at 680 nm from 490 nm.

### Intracellular reactive oxygen species (ROS) assay

Generation of ROS was detected using Fluorometric intracellular ROS kit (Sigma Aldrich, MO, USA) as per manufacturer’s protocol. Fluorescence was measured at excitation and emission of 650 nm and 675 nm respectively via multimode plate reader^50,51^.

### Griess assay

Nitric oxide (NO) release was estimated using the Griess assay. Briefly, after 24 h of drug treatment, Griess reagent (Sigma Aldrich, MO, USA) was added to cells in equal volume and incubated for 30 min in the dark, and absorbance was taken at 540 nm via multimode plate reader^23^.

### Fura-2AM assay

Intracellular calcium flux was measured using the fluorescent radiometric Ca^2+^ indicator, Fura-2 acetoxymethyl ester (Fura-2, AM; Invitrogen, Carlsbad, USA). Briefly, after 24 h of treatment, Fura-2AM (1 μmol/L) was added to cells and incubated for 30 min at 37 °C. The intracellular calcium flux was measured at excitation of 340 nm and 380 nm and emission of 510 nm using a multimode plate reader.

### TMRE assay

Mitochondrial membrane potential of the cells after drug treatment was detected using tetramethylrhodamine ethyl ester dye (TMRE; Molecular Probes, CA, USA). The cells were incubated with 0.3 µM TMRE dye and incubated at 37 °C for 30 min in dark. Fluorescence intensity of TMRE was measured at excitation and emission of 549 nm and 574 nm respectively via a multimode plate reader.

### Gene expression analysis

Total ribonucleic acid (RNA) was isolated using RNAiso Plus (TaKaRa, Shiga, Japan) followed by cDNA conversion using PrimeScript™ 1st strand cDNA synthesis kit (TaKaRa, Shiga, Japan). Quantitative reverse transcriptase-polymerase chain reaction (qRT-PCR) was performed by using *TB Green™ Premix Ex Taq™ II* (Tli RNase H Plus; TaKaRa, Shiga, Japan) for cardiac markers in a QuantStudio*™* 5 real-time PCR Systems (Applied Biosystems, USA). All the primers were purchased from Sigma Aldrich, Bangalore, India (Table 1). The relative gene expression was calculated using 2^-∆∆CT^ and GAPDH, a housekeeping gene was used as endogenous control^52,53^.

**Table 1:** List of primers.

Gene	Sequence	Product length (bp)	Annealing temperature (°C)
GAPDH	F—5ʹ-CGACCACTTGTCAAGCTCA-3ʹ	202	59
	R—5ʹ-AGGGGAGATTCAGTGTGGT-3ʹ		
NKX2.5	F—5ʹ-CAAGTGTGCGTCTGCCTTTC-3ʹ	104	54
	R—5ʹ-CGCACAGCTCTTTCTTTTCGG-3ʹ		
GATA4	F—5ʹ-CCTGGAAGACACCCCAATCTC-3ʹ	119	55
	R—5ʹ-AGGTAGTGTCCCGTCCCATCT-3ʹ		
Troponin I	F—5ʹ-CTGCGGAGAGTGAGGATCTC-3ʹ	111	60
	R—5ʹ-GTCCTCCTTCTTCACCTGCT-3ʹ		
Troponin T	F—5ʹ-GCGGAAGAGTGGGAAGAGACA-3′	116	65
	R—5′-CCACAGCTCCTTGGCCTTCT-3′		
MHCα	F—5′-TCCTGCGGCCCAGATTCTTC-3′	264	55
	R—5′-CCGTCTTCCCATTCTCGGTT-3′		

### Statistical analysis

Data were analyzed in GraphPad Prism v8 (GraphPad Software, Inc., CA, USA) and are expressed as mean ± SEM. Student’s t-test or one-way ANOVA followed by Dunnett’s post-test was performed. Data were considered statistically significant when p < 0.05.

## Results

### Generation of cardiomyocytes from WJMSCs

**Figure 1:**
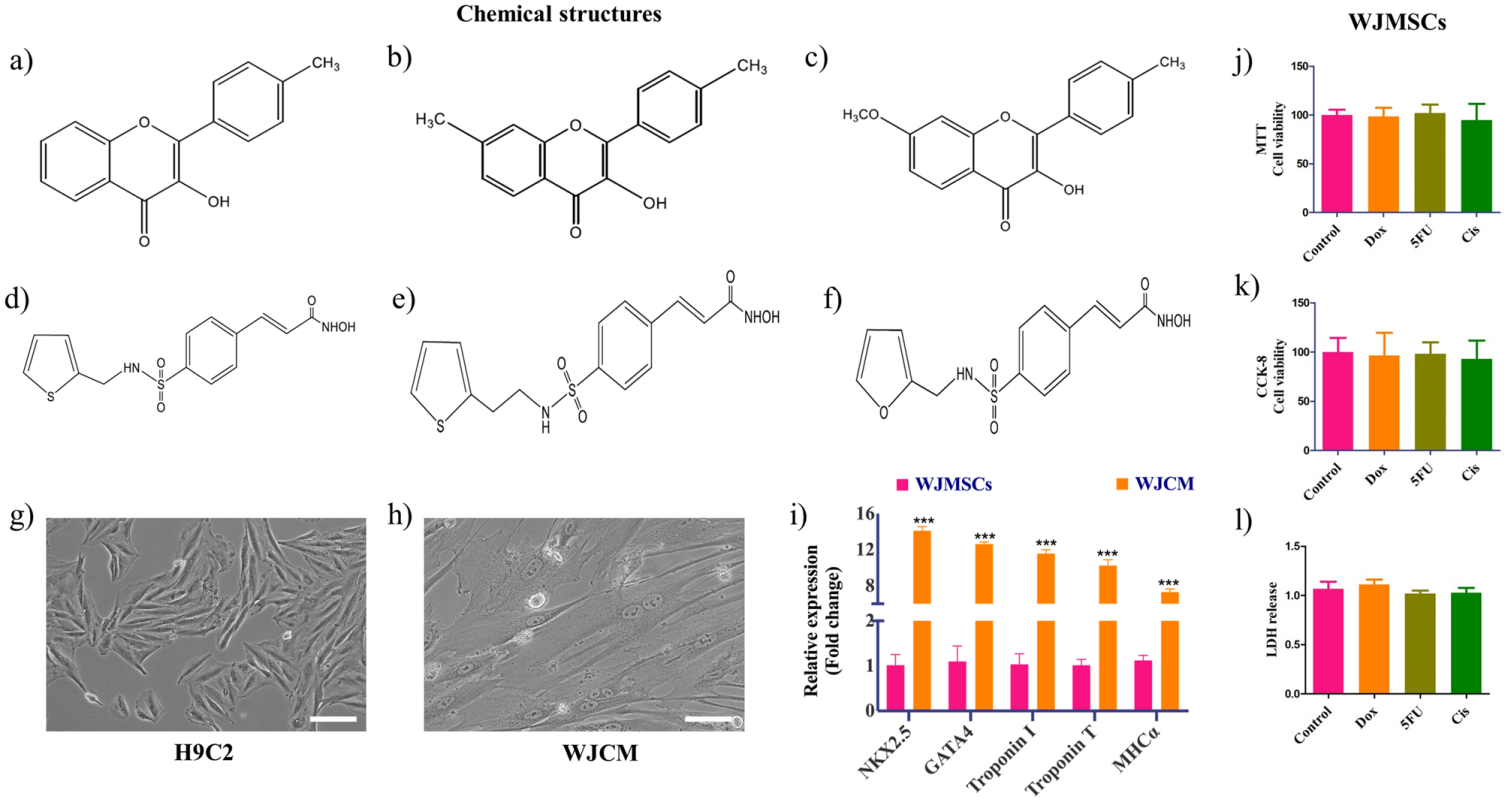
Culture of H9C2 cells and cardiomyocytes derived from WJMSCs (WJCM) and its characterization. The chemical structure of the analogues of quercetin (**a**) QMJ-2 (Chemical Name: 3-hydroxy-2-(4-methylphenyl)-4H-1-benzopyran-4-one), (**b**) QMJ-5 (Chemical Name: 3-Hydroxy-7-methyl-2-(4-methylphenyl)-4H-1-benzopyran-4-one), (**c**) QMJ-6 (Chemical Name: 3-Hydroxy-7-methoxy-2-(4-methylphenyl)-4H-1-benzopyran-4-one), and cinnamic acid (**d**) NMJ-1 (Chemical name: (E)-NHydroxy-3-(4-(N-(thiophen-2yl)methyl)sulfamoyl)phenyl)acrylamide), (**e**) NMJ-2, (Chemical Name: (E)-NHydroxy-3-(4-(N-(thiophen-2yl)ethyl)sulfamoyl)phenyl)acrylamide) (**f**) NMJ-3 ((E)-NHydroxy-3-(4-(N-(furan-2yl)methyl)sulfamoyl)phenyl)acrylamide). Photomicrographs of (**g**) H9C2 cells and, (**h**) WJMSCs differentiated into cardiomyocytes (WJCM). (**i**) qRT-PCR analysis shows an increase in the gene expression of cardiac markers, NKX2.5, GATA4, Troponin I, Troponin T, and MHC-alpha in WJCM. Scale bar 50 μm. The cytotoxicity assays (**j**) MTT cell viability, (**k**) cell counting kit-8 (CCK-8), and (**l**) LDH release were performed in WJMSCs. Data represented as mean ± SEM (n = 3), ***p < 0.001. *Dox* doxorubicin, *5FU* 5-fluorouracil, *Cis* cisplatin.

The chemical structure of the analogues of quercetin QMJ-2, QMJ-5, and QMJ-6, and cinnamic acid NMJ-1, NMJ-2, and NMJ-3 used in the study are indicated (Fig. 1a–f). H9C2 cells (Fig. 1g) and WJMSCs were differentiated into cardiomyocytes (WJCM) using 5-azacytidine and Trichostatin A (TSA) (Fig. 1h). Gene expression analysis by qRT-PCR confirmed the presence of cardiac markers NKX2.5, GATA binding protein 4 (GATA4), Troponin I, Troponin T and myosin heavy chain-alpha (MHC⍺) in WJCM as compared to WJMSCs (Fig. 1i). To differentiate the cardiotoxicity from general cell toxicity, we performed MTT (Fig. 1j), CCK-8 (Fig. 1k), and LDH (Fig. 1l) assays in undifferentiated WJMSCs. We treated the WJMSCs with Dox, 5FU, and Cis for 24 h. There was no significant difference observed which suggests that toxicity is selective to WJCM.

### Assessment of quercetin and cinnamic acid analogues on viability and survival of rat cardiomyocytes and WJCM

**Figure 2:**
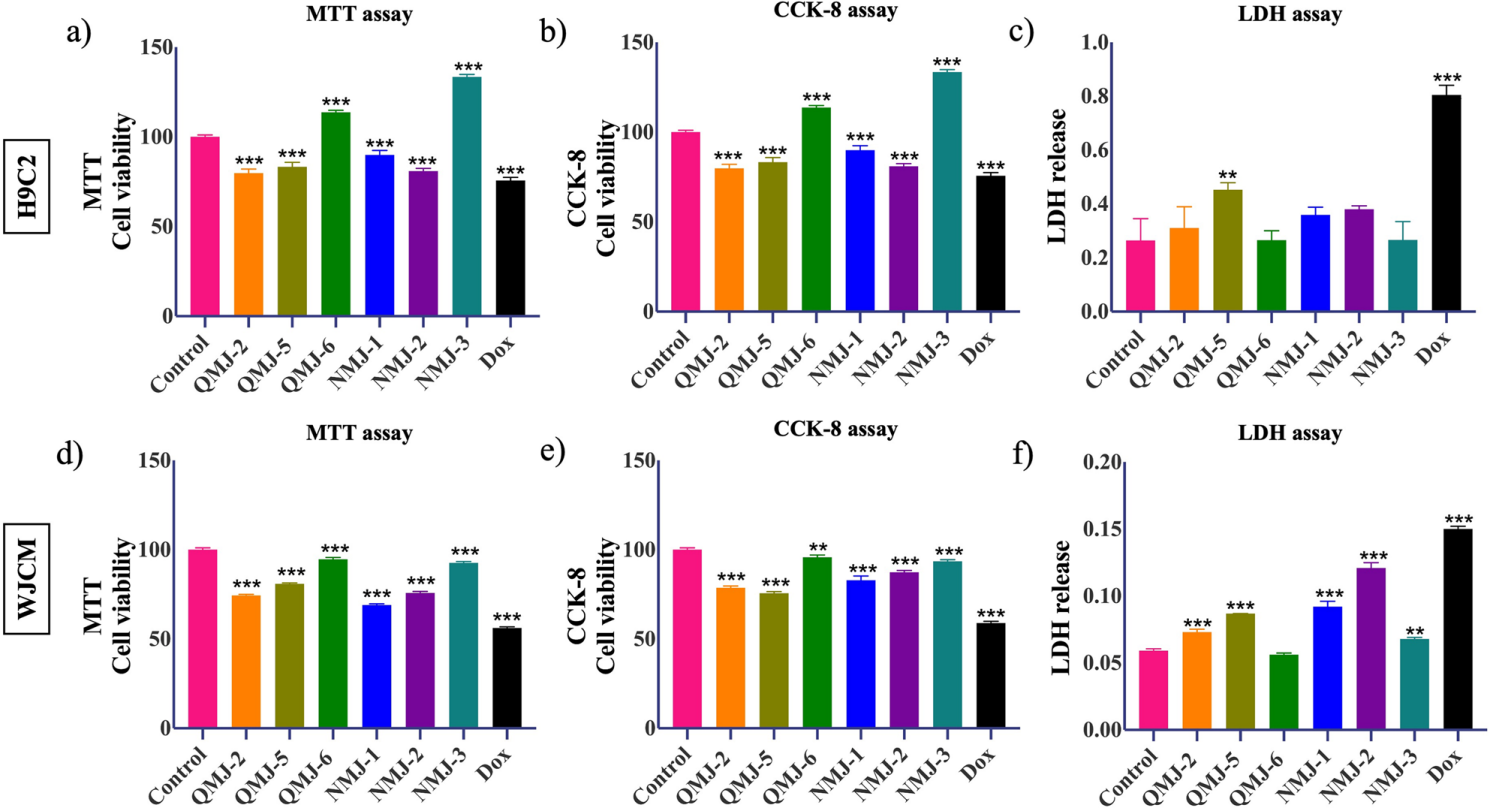
Effect of quercetin and cinnamic acid analogues on cell proliferation and cytotoxicity. H9C2 cells and WJCM after treatment with QMJ-2,-5,-6 or NMJ-1,-2,-3 were assessed for cell viability by using (**a**,**d**) MTT cell viability assay and, (**b**,**e**) cell counting kit-8 assay. (**c**,**f**) Cytotoxic effects of these compounds were estimated in terms of LDH release. Data represented as mean ± SEM (n = 3), *p < 0.033, **p < 0.002, ***p < 0.001. *Dox* doxorubicin.

Cardiotoxicity of quercetin (3-hydroxyflavone; QMJ) and cinnamic acid (cinnamyl sulphonamide hydroxamate; NMJ) analogues were determined by MTT cell viability, CCK-8, and LDH assay. H9C2 cells and WJCM were exposed to drug derivatives or oncotherapeutic, Dox (1 µM) for 24 h. We observed a decrease in viability of H9C2 cells to about 10–20% after treatment with QMJ-2, 5, NMJ-1, and 3, and about a 25% decrease in viability with Dox, the positive cardiotoxic drug control. Surprisingly, QMJ-6 and NMJ-3 showed an increase in viability and were not cardiotoxic (Fig. 2a). We then performed a CCK-8 assay to further confirm these results and obtained similar results (Fig. 2b). These results were comparable to WJCM wherein QMJ-6 and NMJ-3 were less toxic as compared to other QMJ and NMJ analogues whereas Dox treatment reduced cell viability drastically (Fig. 2d,e). We observed that NMJ-2 was also less toxic and similar to NMJ-3 in WJCM (Fig. 2e). To further investigate the effect of quercetin and cinnamic acid analogues, we measured the activity of LDH. In both H9C2 cells and WJCM, an increase in LDH was observed in cells treated with QMJ-2, QMJ-5, NMJ-1, and NMJ-2 which indicates a clear response to cell membrane injury. On the other hand, QMJ-6 and NMJ-3 treatment showed no to little change in LDH release in both H9C2 cells and WJCM (Fig. 2c,f).

### Status of ROS generation and NO release upon treatment with quercetin and cinnamic acid analogues

**Figure 3:**
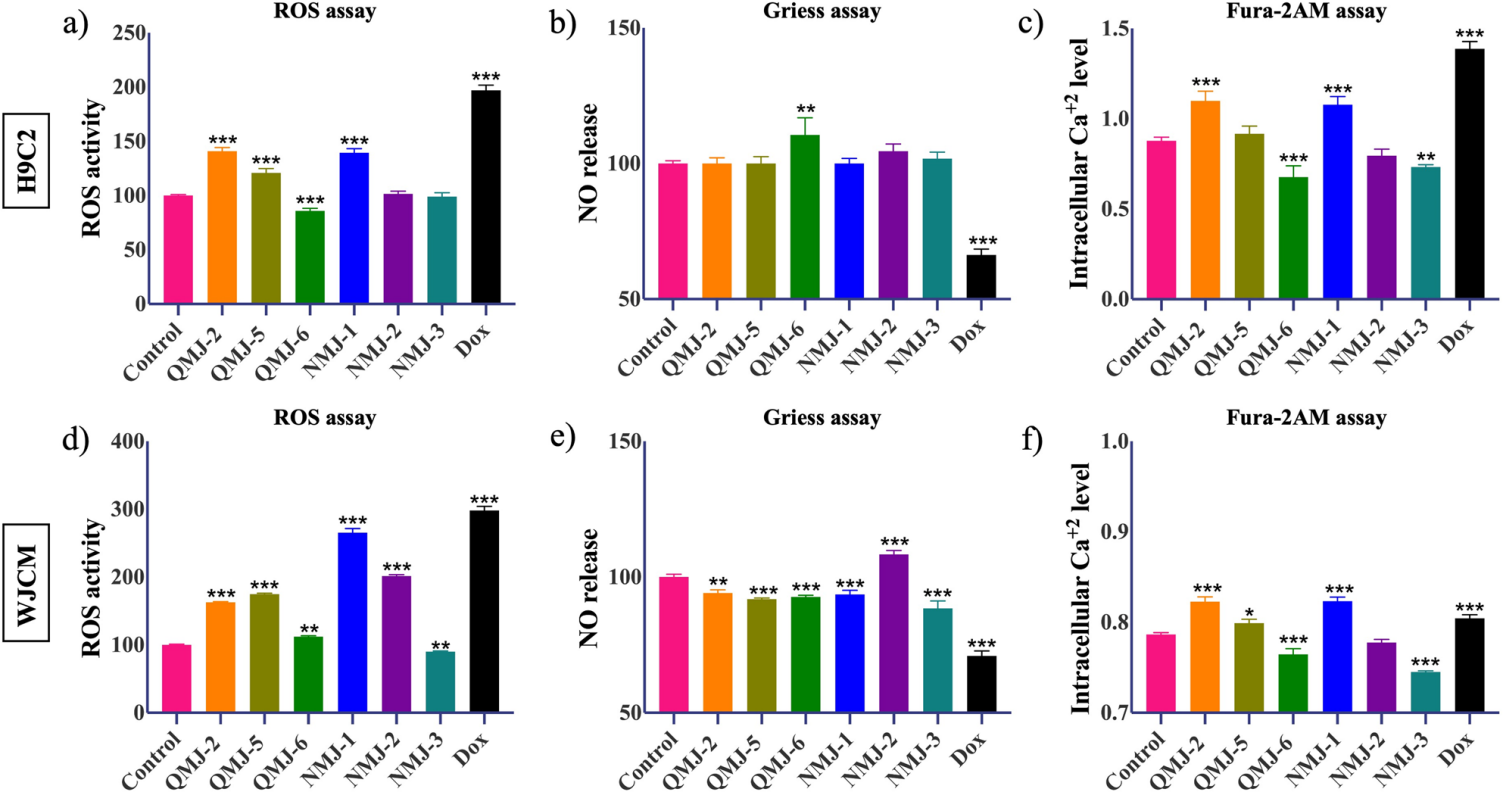
Detection of oxidative stress and intracellular Ca^2+^ flux in H9C2 cells and WJCM. (**a**,**d**) Reactive oxygen species (ROS) activity and, (**b**,**e**) nitric oxide (NO) release by Griess assay were assessed after treatment with quercetin or cinnamic acid analogues for 24 h. (**c**,**f**) Accumulation of calcium after the treatments was estimated by using Fura-2AM dye. Data represented as mean ± SEM (n = 3), *p < 0.033, **p < 0.002, ***p < 0.001. *Dox* doxorubicin.

Excessive reactive oxygen species (ROS) is one of the main sources of cell damage which leads to cardiotoxicity. We thus measured ROS activity in H9C2 cells and WJCM after exposure to the drug analogues. Similar to previous results, both QMJ-6 and NMJ-3 were not toxic to the cells, and along with NMJ-2 in WJCM, there was lowered ROS activity whereas an increase in ROS activity was detected after treatment with the rest of the analogues (Fig. 3a,d). Furthermore, we also estimated the levels of nitric oxide (NO), a known vasodilator^54^ by using a modified Griess reagent. QMJ-6 and NMJ-2 in H9C2 cells (Fig. 3b) and NMJ-2 in WJCM elevated NO levels while NMJ-3 decreased NO level by 12% WJCM (Fig. 3e). Rest of the analogues did not show much effect.

### Cardiotoxic effect of quercetin and cinnamic acid analogues on calcium accumulation

Initiation of myocardial injury and impairment of contractile function is thought to be caused due to accumulation of intracellular Ca^2+^. Here, we observed a decrease in Ca^2+^ accumulation after exposure of cells to QMJ-6, NMJ-2, and NMJ-3 in both H9C2 and WJCM cells (Fig. 3c,f). The other analogues studied displayed cardiotoxicity similar to Dox (in WJCM) or higher calcium accumulation (in H9C2).

### Modulation of mitochondrial membrane potential after treatment with quercetin and cinnamic acid analogues

**Figure 4:**
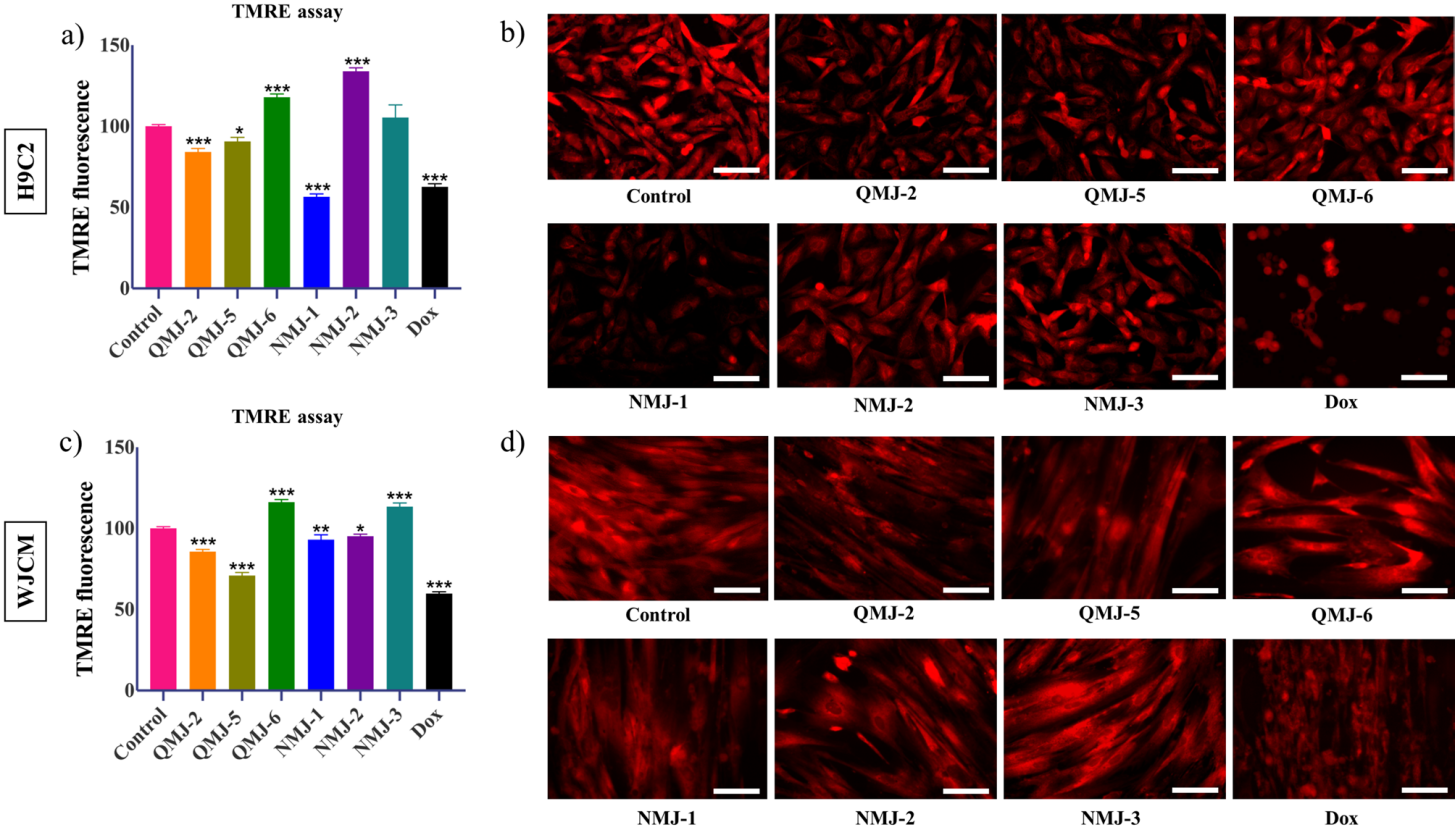
Mitochondrial membrane potential in H9C2 cells and WJCM. (**a**, **c**) Quantitative measurement of fluorescence intensity of cells stained with TMRE dye after treatment with quercetin or cinnamic acid analogues and (**b**, **d**) microscopic images of cells captured under a fluorescence microscope. Data represented as mean ± SEM (n = 3), *p < 0.033, **p < 0.002, ***p < 0.001. Scale bar 50 μm. *Dox* doxorubicin.

Depolarization of mitochondrial membrane and associated ROS production leads to induction of apoptosis in cells. We observed increased mitochondrial membrane potential using TMRE in H9C2 cells treated with QMJ-6, NMJ-2, and NMJ-3 (Fig. 4a,b) and WJCM treated with QMJ-6 and NMJ-3 (Fig. 4c,d) detected quantitatively and by fluorescent microscopy.

### Expression of cardiac markers after treatment with quercetin and cinnamic acid analogues in WJCM

**Figure 5:**
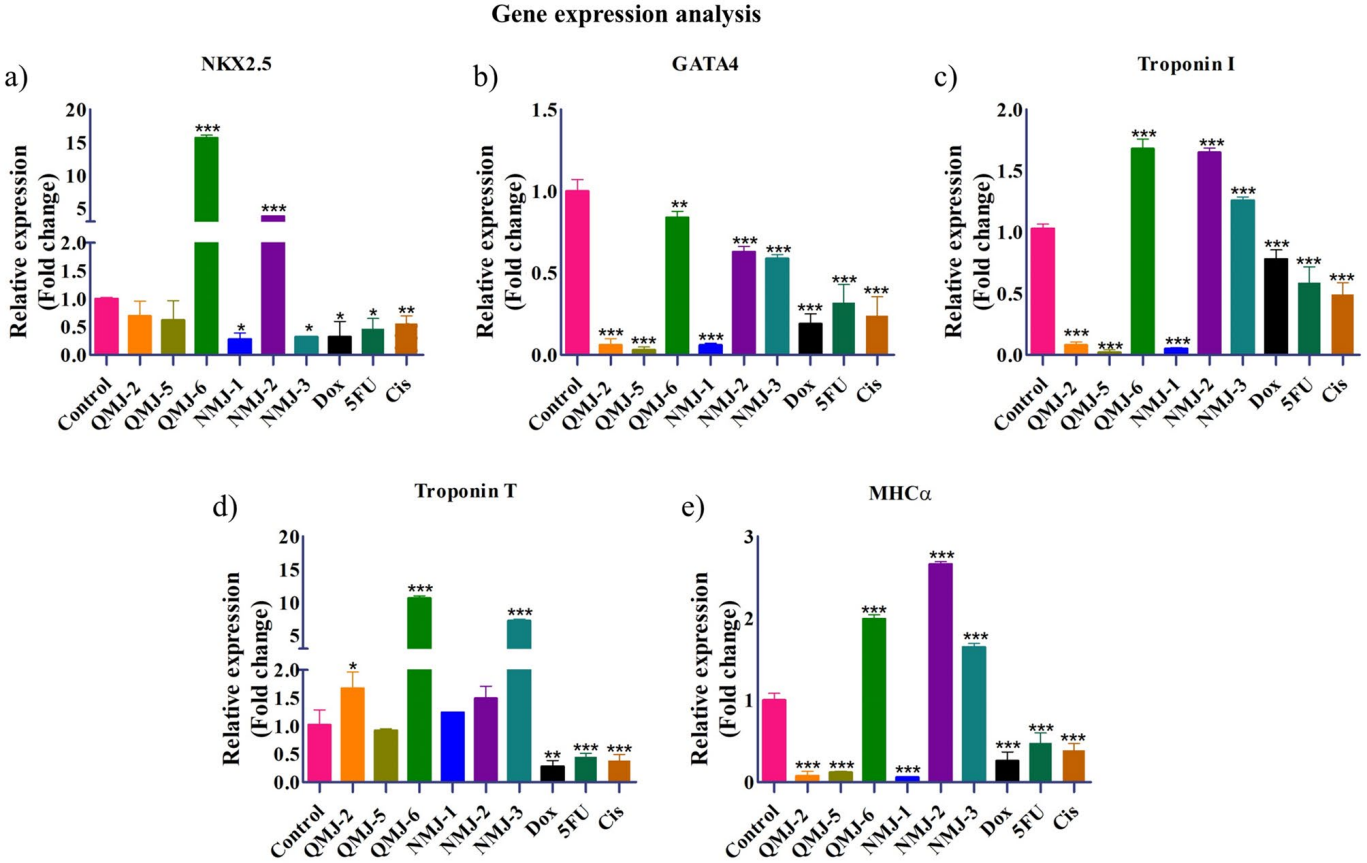
Gene expression analysis by qRT-PCR of cardiac-specific markers. WJCM treated with QMJ-2,-5,-6 and NMJ-1,-2,-3 were analysed for (**a**) NKX2.5, (**b**) GATA4, (**c**) Troponin I, (**d**) Troponin T, and (**e**) MHCα. Data represented as mean ± SEM (n = 3), *p < 0.033, **p < 0.002, ***p < 0.001. *Dox* doxorubicin, *5FU* 5-fluorouracil, *Cis* cisplatin.

To further investigate the cardiotoxic effect of these compounds in human-derived cardiomyocytes, we exposed WJCM with quercetin and cinnamic acid analogues followed by qRT-PCR analysis for cardiac genes. In concordance with the cardiotoxic parameters tested, it was also seen that drug analogues QMJ-2, QMJ-5, and NMJ-1 decreased the expression of cardiac genes GATA-4, Troponin I, and MHC⍺ (Fig. 5b–e). Treatment of QMJ-6 and NMJ-2 caused a significant increase in the expression of NKX2.5 (Fig. 5a) whereas QMJ-6, NMJ-2, and NMJ-3 treated cells saw a marked upregulation of GATA4 (Fig. 5b), Troponin I (Fig. 5c), and MHC⍺ (Fig. 5e). Significantly QMJ-6 and NMJ-3 showed an emphatically higher (8–10 fold) Troponin T gene expression (Fig. 5d) with a milder increase observed with treatment with QMJ-2 and NMJ-2. The treatment of cardiotoxic drugs Dox, 5FU, and Cis reduced all the cardiac markers expression drastically.

## Discussion

For many years, clinical data have substantially demonstrated that cancer drug-induced cardiotoxicity among cancer survivors leads to the loss of cardiomyocytes which ultimately compromises heart function^55^. There is a need for testing the hidden cardiotoxicity of any drug molecule before administering it to patients which will also increase the success rate of the drug marketing process. We thus need a suitable human-based model to test the cardiotoxicity. In this present study, we have established a cardiomyocytes model derived from human WJMSCs and assessed the utility of cardiomyocytes in monitoring the cardiotoxic events of novel anticancer drug molecules at the cellular and functional level. The advantage of using WJMSCs is that it is isolated using a non-invasive method and from a surgically discarded tissue. We have optimized an easy differentiation procedure to generate cardiomyocytes. These differentiated cells can be further cultured and maintained in a hassle-free manner.

Our lab has previously reported the isolation of WJMSCs and their differentiation into functional cardiomyocytes^23,53^. Along with primary cardiomyocytes, based on the characteristics features, we considered H9C2 cells to understand and compare the impact of cardiotoxicity in an immortalized cell line with primary differentiated cardiomyocytes. For instance, the presence of cytoskeletal protein, beta-tubulin II, and their interactions with mitochondria is very crucial for energy metabolism in the heart. Kuznetsov et al.^32^ attempted to study the difference in bioenergetics, and metabolic and functional characteristics between HL-1 (adult immortalized atrial cells) and H9C2 cells. It was found that beta-tubulin II was absent in HL-1 but present in H9C2 cells which suggest that H9C2 cells are bioenergetically/biochemically similar to primary cardiomyocytes. The heart requires an extensive amount of energy to carry out the function of blood pumping and 80% of the energy is supplied by mitochondria. The study revealed that the H9C2 cell has a content of mitochondrial mass, increased respiration rate, and enhanced ATP level in comparison to HL-1 cells^32^.

We investigated the effects of 3-hydroxyflavone (QMJ-2, QMJ-5, and QMJ-6) and cinnamyl sulfonamide-hydroxamate (NMJ-1, NMJ-2, NMJ-3) in WJCM and H9C2 cells using the concentrations reported for non-cancerous Vero cells. The prerequisite for any synthesized drug molecules is that they should be nontoxic to cardiac cells. We could show by MTT, CCK-8, and LDH assay that QMJ-6, NMJ-2, and NMJ-3 had no cytotoxic effect against cardiomyocytes whereas QMJ-2, QMJ-5, and NMJ-1 had induced toxicity suggesting that QMJ-2, QMJ-5, and NMJ-1 that these drugs could have an off-target cardiotoxic effect in vivo.

The cancer drug-induced toxicity in patients is still an unavoidable clinical hurdle. The root of the problem is at the cellular level when chemotherapeutic drugs fail to differentiate between normal cells and tumor cells and indiscriminately target the normal cells causing undesired cytotoxicity. Therefore, a representative in vitro toxicity model is needed to understand the difference between target and non-target cells and then predict the possible toxicity^56^.

In our study, we checked the ability of our model to sense the difference between target and non-target cells, we performed cytotoxicity assays in undifferentiated Wharton’s jelly mesenchymal stem cells considering them as normal cells to test the effect of chemotherapeutic drugs. We treated the WJMSCs with Dox, 5FU, and Cis at the same concentration used for cardiomyocytes. Interestingly, we found that these drugs do not exert any toxicity to WJMSCs and it shows that the model predicting the toxicity is specific to cardiac cells. Furthermore, studying primary cardiomyocytes provides many inherent benefits over whole-heart models whereas H9C2 cells have been the workhorse of in vitro cardiotoxic studies, both offering thorough control of experimental conditions including functionality, bioenergetics, metabolism, and genetic manipulations.

ROS at low amounts acts as “redox messengers” in the proliferation, survival, and differentiation of the cell. Meanwhile, disruption of mitochondria produces high ROS that ultimately becomes a reason for apoptosis^57^. During cardiotoxicity, elevated ROS alters physiological signaling responses which cause cellular hypertrophy, ventricular remodeling, sarcomeric instability, and calcium homeostasis, all these phenomena eventually induces cardiomyocytes death^58^. ROS generation damages the DNA and reduces the DNA binding activity of GATA binding protein 4 (GATA4), a critical cardiomyocytes transcriptional factor^59^. Our study showed that the ROS level remained unaffected in QMJ-6, NMJ-2, and NMJ-3 treated cardiomyocytes, however, QMJ-2, QMJ-5, and NMJ-1 treatment increased ROS levels that ultimately reduced cell viability. Dox generates ROS as a prime mechanism to induce toxicity against cardiomyocytes^60^. In the present study, increased ROS level in dox treatment is in agreement with the established mechanism.

Nitric oxide is a vasodilator, modulator of blood pressure, vascular tone, and hemodynamics^61^ and NO addition promoted differentiation of embryonic stem cells into myocardial cells^62,63^. Production of NO gets altered by the binding of Dox leading to superoxide formation^10^. In our studies, we could demonstrate that Dox addition had reduced NO production, however, the synthesized compounds have not caused any changes in NO levels which indicates that NO physiology remains unaffected.

Cardiotoxicity induces dysregulation of intracellular calcium levels within cardiac cells that imply a cardiac remodeling. The toxicity suppresses mitochondrial respiration causing a low ATP state, which in turn slows the sarcoplasmic reticulum Ca^2+^-ATPase (SERCA) which causes an increase in diastolic potential. This change in intracellular Ca^2+^ homeostasis is due to Ca^2+^ unloading from the sarcoplasmic reticulum^64^ which might lower the heart systolic potential and increase the chance of sudden cardiac arrest^65–67^. In our study, we observed that the intracellular calcium level remained low in QMJ-6, NMJ-2, and NMJ-3 treated cardiomyocytes, whereas QMJ-2, QMJ-5, and NMJ-1 exposure has increased the level indicating dysregulation of calcium homeostasis. Dox promotes the calcium accumulation in the mitochondria via the opening of mitochondrial permeability transition pores which alters the calcium balance^68^. Our result confirms the alternation in calcium due to Dox treatment.

Mitochondrial-mediated cell death has emerged as a major mechanism for the death of cardiomyocytes. Cardiotoxicity disrupts mitochondria and their membrane potential which generates oxidative stress causing excessive ROS production in cardiomyocytes^58^. Upon treatment of cardiomyocytes with QMJ-6, NMJ-2, and NMJ-3, we observed that active mitochondria status remains unaffected whereas, in QMJ-2, QMJ-5, and NMJ-1 treatment cardiomyocytes displayed a loss of mitochondria. However, Dox treatment affected cardiomyocyte mitochondrial activity in concordance with the previous report^60^.

Cardiotoxicity drastically impacts the expression of cardiac genes such as NKX2.5, GATA4, cardiac troponins I and T, and myosin heavy chain alpha which are the functional markers of cardiomyocytes and can be used to assess cardiomyocyte's health and predict cardiac injury^69,70^. Troponins are the gold standard biomarkers that appear in blood with an increase in level within 2–3 h after myocardial damage due to cardiotoxicity^71^. A clinical study found various troponin I release pattern in breast cancer patients before and after chemotherapy where the major number of patients after chemotherapy had increased troponin I level suggesting that they could have a higher incidence of adverse heart effects and a greater left ventricular ejection fraction reduction as compared with patients who had little increase in troponin I level^72^. In the current study, using WJCM, we could see that similar other parameters assessed for cardiotoxicity, QMJ-2, QMJ-5, and NMJ-1 had a drastic reduction in the cardiac marker gene expression, similar to the cardiotoxic effect of Dox, 5FU, and Cis whereas QMJ-6, NMJ-2, and NMJ-3 had no significant impact on cardiac-specific genes. Thus, it is evident that across the parameters analyzed, quercetin and cinnamic acid derivatives QMJ-2, QMJ-5, and NMJ-1 are cardiotoxic, similar to the known cardiotoxic oncotherapeutics Dox, 5FU, and Cis whereas derivatives QMJ-6, NMJ-2, and NMJ-3 do not display significant cardiotoxicity. Furthermore, the compounds QMJ-2, QMJ-5, and NMJ-1 at a concentration of 68 μM, 27.4 μM, and 5.07 μM respectively found effective against cancer could be also actually toxic to cardiomyocytes. We considered the concentrations based on previously reported studies. In the previous studies, the in vitro cytotoxicity assay in Vero cells revealed that the treatment of derivatives QMJ-2, QMJ-5, QMJ-6, NMJ-1, NMJ-2, and NMJ-3 at concentrations of 140 µM, 55.6 µM, 153 µM, 9.7 µM, 8.2 µM, and 15.1 µM respectively reduces the cell viability by 50%. Vero cells are one of the most commonly used mammalian cell lines to study drug efficacy. Given that, we used the same concentrations to test the cardiotoxic effect in our model. Our observation suggests that the cardiotoxicity was specific to the derivative and not an outcome of concentrations. For example, NMJ-1 which was of quite low concentration i.e., 9.7 µM showed cardiotoxicity whereas QMJ-6 at a concentration of 153 µM was found non-cardiotoxic. The data obtained thus justified the purpose of the model which is to detect the cardiotoxicity exerted by any compound and the results are highly comparable with positive controls. To reduce the cardiotoxicity of these analogues, optimum concentration will have to be fixed which is effective against cancers and is not toxic to cardiomyocytes.

The analogues QMJ-2, QMJ-5, and QMJ-6 are the result of substitutions in the 3-hydroxyflavone, a backbone of all flavonols whereas NMJ-1, NMJ-2, and NMJ-3 have been obtained after the modification based on bio-isosterism in the cinnamic acid moiety. Specifically, QMJ-2, QMJ-5, and QMJ-6 are 3-hydroxyflavone analogue with a methyl, di-methyl, and methyl and a methoxy substitution on the phenyl ring respectively^44^. NMJ-1, NMJ-2, and NMJ-3 are cinnamyl sulfonamide hydroxamate derivative with thiophene methyl amine, thiophene ethyl amine, and furfurylamine substitution respectively^43,45^. With various fundamental medicinal chemistry principles such as bio-isosterism, a wide range of chemical compounds are being synthesized with the purpose to augment absorption, distribution, metabolism, excretion, and toxicity (ADMET), improving efficacy, and enhancing chemical accessibility^73^. Fluorouracil is itself a chemical modification^74^. Furthermore, to overcome the issue of drug resistance and cardiotoxicity, Dox^75^ has undergone several structural modifications, and new drug entities such as epirubicin, pirarubicin, valrubicin, and idarubicin have been synthesized^73^. The chemical modification brings significant therapeutic consequences and makes the lead compound highly relevant biologically^76^. In the Dox structure, the lipophilic part demonstrated acidic and hydrophilic amine sugar part showed basic nature which makes them soluble in both lipophilic and hydrophilic types of solvents so they bind to the plasma protein and cell membrane. Upon entering into the cells, Dox intercalates into nucleic acids and inhibits topoisomerase II which explains the mode of action. During the cell-killing action, excessive calcium release and dysregulation of mitochondrial function leads to the undesired outcomes^77,78^. Similarly, the analogues used in this study went through different chemical substitution with methyl and amine groups which may impact the molecular lipophilicity and cellular uptake and these could be possible reasons behind the cardiotoxicity as an off-target effect. However, it would be very interesting to study further the association of structural modification and its off-target effect.

A limitation of the study is that we have only assessed three chemotherapeutic drugs (Doxorubicin, 5-fluorouracil, and cisplatin). There are other classes of chemotherapeutic drugs which exerts cardiotoxicity, as well as various potential natural compounds which may have hidden cardiotoxic effect and so analysing their cardiotoxicity in our model, might have given new prospect. To establish the cardiotoxic effect, we have included an established cell line, H9C2 cells for comparison. Likewise, iPSCs-derived cardiomyocytes have been widely used to study cardiotoxicity. An inclusion of iPSCs would have made the comparison more exhaustive, however, iPSCs hold a set of challenges for disease modelling including cardiovascular diseases^79,80^. It has been observed that experimental and genetic variability in iPSCs can be overcome up to a certain limit but iPSCs culturing and maintenance is expensive and remains out of budget for many research labs across the world. Regarding animal studies, because of shortcomings like high cost, interspecies variability, and ethical concerns, the demand for an alternative to animal use is rising^14^.

In our study, we tested synthetic compounds which have already shown anti-cancer effects. Our model further indicates that the concentrations of few compounds which exerted anticancer effect could possibly show cardiotoxic effect too. We included experiments to study cardiotoxicity which result in cardiomyocyte death which is mainly mitochondria-mediated apoptosis. We believe that our model and experiments can be used to study hidden cardiotoxicity of other synthesized compounds, natural compounds, drugs molecules, etc.

## Conclusion

In summary, by studying the oncotherapeutic molecules in human stem cells-derived cardiomyocytes we could effectively ascertain the varying cardiotoxic response of these molecules. From a perspective of human origin, human stem cells-derived cardiomyocytes could be a perfect preclinical cell-based model to study cardiotoxic events and reduce the dependence on animal usage in drug safety research.

## Data Availability

The datasets used and/or analyzed during the current study available from the corresponding author on reasonable request.
